# Functional and radiographical results of asymmetrically reconstructed total hip arthroplasty in patients with bilateral dysplastic arthritic hips with one hip Crowe II–III and the other Crowe IV: a retrospective cohort study

**DOI:** 10.1186/s10195-021-00576-w

**Published:** 2021-03-13

**Authors:** Junmin Shen, Jingyang Sun, Yinqiao Du, Bohan Zhang, Tiejian Li, Yonggang Zhou

**Affiliations:** 1 Medical School of Chinese People’s Liberation Army, Beijing, 100853 China; 2 Department of Orthopedics, The First Medical Centre, Chinese People’s Liberation Army General Hospital, 28 Fuxing Road, Haidian District, Beijing, 100853 China

**Keywords:** High hip center, Developmental dysplasia of the hip, Crowe II–III, Crowe IV, Total hip arthroplasty

## Abstract

**Background:**

The study aimed to evaluate the functional and radiographical results of asymmetrically reconstructed total hip arthroplasty in patients with bilateral dysplastic arthritic hips with one hip Crowe II–III and the other hip Crowe IV.

**Materials and methods:**

From April 2006 to April 2019, we evaluated 23 patients who had a reconstruction of one Crowe II–III hip with high hip center (HHC) and the other Crowe IV hip at the anatomical position (H group). The radiographic and clinical outcomes were compared with those of a control group of 19 patients with bilateral dysplasia who had one Crowe IV hip and the contralateral hip both reconstructed in the anatomical position (A group). Medical records and radiographs were reviewed, and a complete follow-up was conducted for all patients.

**Results:**

The mean vertical center of rotation (V-COR) and horizontal center of rotation (H-COR) in the H group were 30.6 ± 5.8 mm and 30.0 ± 5.5 mm, respectively. In the A group, the corresponding values were 14.0 ± 4.3 mm and 23.0 ± 2.3 mm, respectively. A significant difference was found in terms of V-COR and H-COR between the two groups, and no significant difference was shown regarding the cup inclination, abductor lever arm (ALA), ALA ratio, and leg length discrepancy (LLD). Three patients of the H group and four patients of the A group exhibited LLD > 10 mm. All seven patients who had LLD > 10 mm underwent the shortening subtrochanteric osteotomy (SSTO) of the Crowe IV hip. Subgroup analysis based on the presence and absence of SSTO showed that the LLD of the SSTO group was greater than that of the non-SSTO group in both groups, but the difference was only statistically significant in the A group. At the last follow-up, the mean Harris Hip Scores significantly improved in the two groups, and there was no revision during the follow-up period. In the H group, four patients presented with a slight limp and three patients with a moderate limp, while it was six patients and one patient in the A group, respectively.

**Conclusions:**

Asymmetrical reconstruction in patients with bilateral dysplastic arthritic hips with one hip Crowe II–III and the other Crowe IV is acceptable and comparable when compared with bilateral anatomical reconstruction.

**Level of evidence:**

III, retrospective observational study.

*Trial registration* Chinese Clinical Trail Registry. ChiCTR2000033848

## Introduction

Total hip arthroplasty (THA) is a prevalent and efficacious procedure for secondary osteoarthritis in patients with developmental dysplasia of the hip (DDH) [1]. The morphological abnormalities of Crowe II–III hips, mainly including the segmental deficiency in the superior and posterosuperior directions of the acetabulum, bring challenges to surgeons during THA [2]. To solve the problem of inadequate bone–implant contact, several treatment options and techniques have been developed, including bulk femoral head autografts, metal augments, acetabular medial wall osteotomy, and high hip center (HHC) technique [3–6].

On account of the advantages of shortening surgical time and simplifying the procedure, HHC was accepted as a valuable and effective alternative. In recent years, for patients with unilateral DDH, many encouraging mid- to long-term results have been reported in the literature [7, 8]. Nawabi et al. [9] reviewed 32 patients with Crowe II–III dysplasia who were treated with HHC at a mean follow-up of 12 years, revealing Kaplan–Meier survivorship for all-cause revisions of 97%. Montalti et al. [10] evaluated 84 THAs with high cup placement, showing overall survivorship of 90.5% at 15 years. There were also several studies of bilateral HHC utilized in dysplastic patients. Through a gait analysis in patients with bilateral DDH, Karaismailoglu et al. [11] concluded that bilateral HHC can give rise to similar gait characteristics as anatomical reconstruction. Shen et al. [8] evaluated 16 patients (32 hips) treated with bilateral HHC and for whom no revision occurred at the last follow-up. However, for patients with bilateral dysplasia who had asymmetrical reconstruction of two hips, the result remains uncertain. Generally, a Crowe IV hip is supposed to be reconstructed in the anatomical position. Thus, for patients with bilateral dysplastic arthritic hips with one Crowe II–III hip in high hip center and the other hip Crowe IV in the anatomical position, the imbalance of the center of rotation on the two sides may affect the restoration of leg length discrepancy (LLD), postoperative gait, and the longevity of prostheses.

Therefore, in this study, we sought to evaluate the functional and radiographical results of asymmetrically reconstructed THA in patients with bilateral dysplastic arthritic hips with one hip Crowe II–III and the other Crowe IV.

## Material and methods

This retrospective study was approved by our institutional ethics review board. From April 2006 to April 2019, we reviewed consecutive 174 patients who were diagnosed with bilateral DDH and underwent cementless THA. From this initial group, we included those patients with reconstruction of one Crowe II-III hip with high hip center and the other Crowe IV hip at the anatomical position as the high hip center group (H group). In addition, we set up an anatomic group (A group) as a comparison, consisting of patients with bilateral dysplasia who had one Crowe IV hip and the contralateral hip both reconstructed in the anatomical position. HHC was defined as 22 mm above the interteardrop line in this study [12]. In the H group, for Crowe IV hip, shortening subtrochanteric transverse osteotomy (SSTO) was sometimes inevitable, but for HHC hip, it was not required. Thus, we excluded patients who were classified as bilateral Crowe IV DDH and treated with bilateral SSTO from the A group. Finally, 23 patients were included in the H group, and 19 patients were included in the A group. A complete follow-up including radiographical and clinical evaluation was available for all patients. Table 1 summarizes the demographic characteristics of the two groups.

**Table 1:** Demographics data

Variable	H group	A group	*p* value
Number of patients	23	19	
Female/male	21/2	19/0	0.492
Age (years)^a^	50.8 ± 10.3 (34–69)	40.4 ± 10.2 (22–60)	0.002
BMI (kg/m^2^)^a^	24.3 ± 4.3 (15.2–32.2)	22.1 ± 2.3 (17.6–28.0)	0.050
Mean follow-up (years)^a^	5.8 ± 3.3 (1.3–12.0)	6.4 ± 3.8 (1.7–14.7)	0.604
Crowe classification (contralateral hips were all type IV)			
I	0	3	
II	7	0	
III	16	13	
IV	0	3	

^a^Values given as mean ± standard deviation (range); BMI, body mass index

### Preoperative planning

Anteroposterior (AP) radiographs of the pelvis were taken preoperatively for every patient. Computer tomography (CT) scan and three-dimensional (3D) reconstruction were performed when an AP radiograph was insufficient to assess the acetabular bone stock. For Crowe II–III hips, if the roof of the true acetabulum was relatively intact and adequate to support the implant, we would decide to insert the cup at the anatomical position; if not, we would use the high hip center technique.

### Surgical technique

All operations were performed under general anesthesia using a posterolateral approach by one senior orthopedic surgeon. All patients were operated bilaterally in one stage. According to the preoperative anteroposterior radiographs, we always first performed the procedure for the hip with higher dislocation height.

For all Crowe IV hips, the cup was placed at the inferior and medial part of the true acetabulum [13]. Because hip reduction with a femoral trial stem was difficult, the SSTO was performed in 15 Crowe IV hips of the H group and 10 Crowe IV hips of the A group. The procedure of SSTO has been described in detail in our previous studies [14]. The mean osteotomy length was 3.8 cm (range 3–5 cm) in the H group while it was 3.66 cm (range 2–5 cm) in the A group. Aiming to facilitate the procedure of hip reduction and simultaneously maintain proper soft tissue tension, the osteotomy length of SSTO was only determined by the distance between the center of the trial femoral head and the center of the acetabular cup during the hip reduction with a femoral trial stem. For Crowe II–III hips of the H group, the HHC technique was used to improve bone–implant contact so that steady initial fixation could be achieved. Deeper reaming through the medial wall was performed to medialize the cup during the preparation of acetabulum. For Crowe III hips of the A group, to insert the cup in the anatomical position, the autogenous femoral head bone graft was applied to provided additional support in three cases. The type and size of implants and bearing are presented in Table 2.

**Table 2:** Type and size of implants and bearing in all hips

	H group		A group	
	H hip	Crowe IV	A hip	Crowe IV
Median cup size (mm) (IQR)	48 (44, 50)	44 (44,46)	44 (44,46)	44 (44,46)
The head diameter				
22 mm	0	0	1	1
28 mm	15	23	16	18
32 mm	5	0	1	0
36 mm	3	0	1	0
Cup type				
Pinnacle (DePuy, Warsaw, IN, USA)	14 (60.9%)	16 (69.6%)	5 (26.3%)	5 (26.3%)
Duroloc (DePuy)	6 (26.1%)	7 (30.4%)	14 (73.7%)	14 (73.7%)
Betacup (Link, Hamburg, Germany)	3 (13.0%)	0 (0%)	0 (0%)	0 (0%)
Femoral stem				
S-ROM (DePuy)	17 (73.9%)	23 (100%)	19 (100%)	19 (100%)
Sleeve	17	18	16	13
Cone	0	5	3	6
Corail (DePuy)	5 (21.7%)	0 (0%)	0 (0%)	0 (0%)
LCU (Link)	1 (4.4%)	0 (0%)	0 (0%)	0 (0%)
Bearing surface				
COC	22 (95.6%)	23 (100%)	18 (94.7%)	18 (94.7%)
COP	1 (4.4%)	0 (0%)	0 (0%)	0 (0%)
MOP	0 (0%)	0 (0%)	1 (5.3%)	1 (5.3%)

IQR, interquartile range; COC, ceramic on ceramic; COP, ceramic on polyethylene; MOP, metal on polyethylene

### Radiographic and clinical assessment

**Fig. 1:**
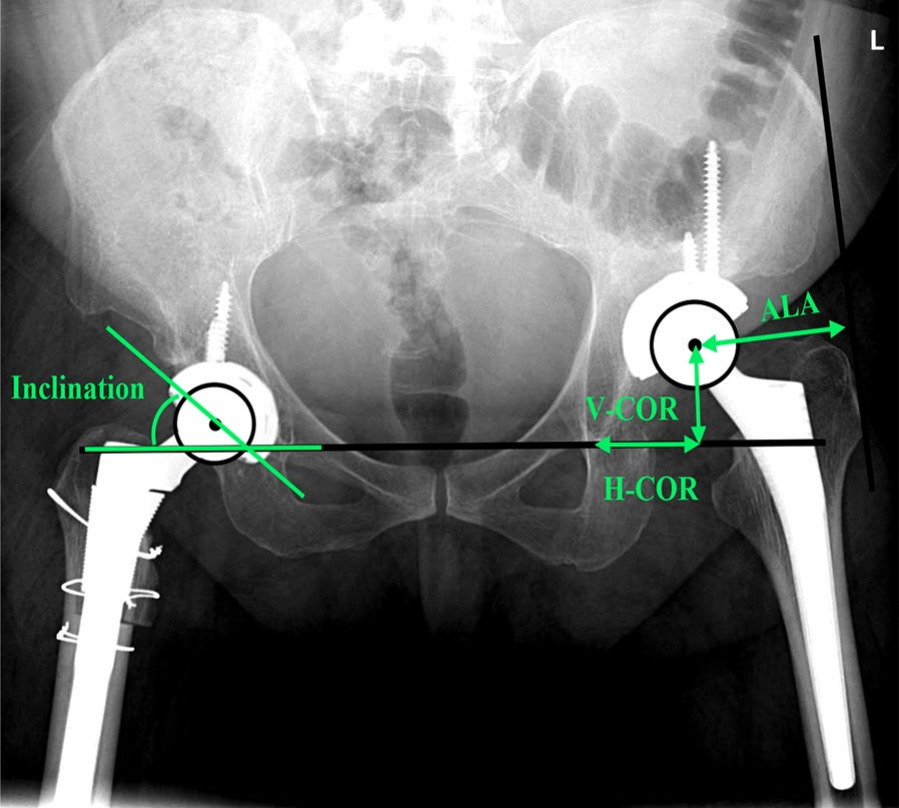
Diagram for postoperative radiographic measurement. V-COR: vertical center of rotation; H-COR: horizontal center of rotation; ALA: abductor lever arm

All patients underwent routine radiographs, including an AP view of the pelvis in the supine position, a lateral view of the affected hip, and a full-length standing AP radiograph. The location of the hip center was determined by the vertical center of rotation (V-COR) and the horizontal center of rotation (H-COR) (Fig. 1). The vertical shift (V-shift) and horizontal shift (H-shift) were defined as the differences in V-COR and H-COR between the two hips [15]. The cup inclination was defined as the abduction angle, formed by the interteardrop line and the connecting line to the edges of the rim of the cup. The abductor lever arm (ALA) was measured from the femoral head to the line joining the lateral part of the greater trochanter to the anterosuperior iliac crest, and the ratio of ALA was calculated (Fig. 1). Leg length was measured as the distance from the teardrop to the center of the ankle joint, and the postoperative leg length discrepancy was calculated [14]. The cup was considered loosened if there was a change > 3 mm of migration or > 4° in the angle of abduction [16].

Clinical and radiographic data were obtained before surgery and at final follow-up. Clinical functional assessment was performed using the Harris Hip Score (HHS). The presence of a positive Trendelenburg sign and limp was recorded. Any visual evidence of a lateral imbalance in the pelvic movement during gait was scored as a limp and was categorized as slight, moderate, or severe [17]. Patient satisfaction was investigated and was subjectively described as excellent, good, moderate, or unsatisfactory.

### Statistical assessment

Differences in radiographic parameters, demographics data, and pre- and postoperative HHS between the two groups were assessed by Student’s *t*-test. Categorical data were compared using a χ^2^-test, and the Mann–Whitney *U* test was used to assess patient satisfaction. All tests were performed using SPSS version 25.0 (IBM, Armonk, NY, USA). Significance was set at *p* < 0.05.

## Results

### Radiographic evaluation

**Fig. 2:**
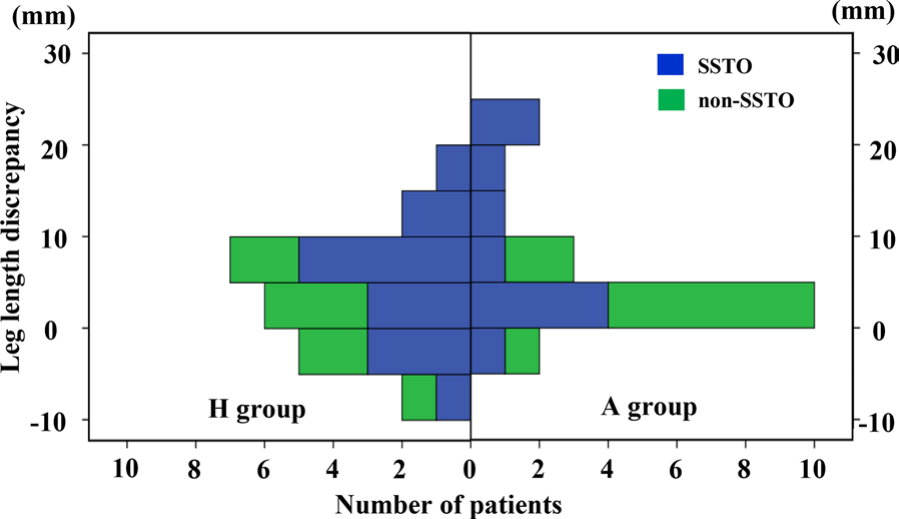
Distribution of LLD in both groups. SSTO, shortening subtrochanteric osteotomy

**Fig. 3:**
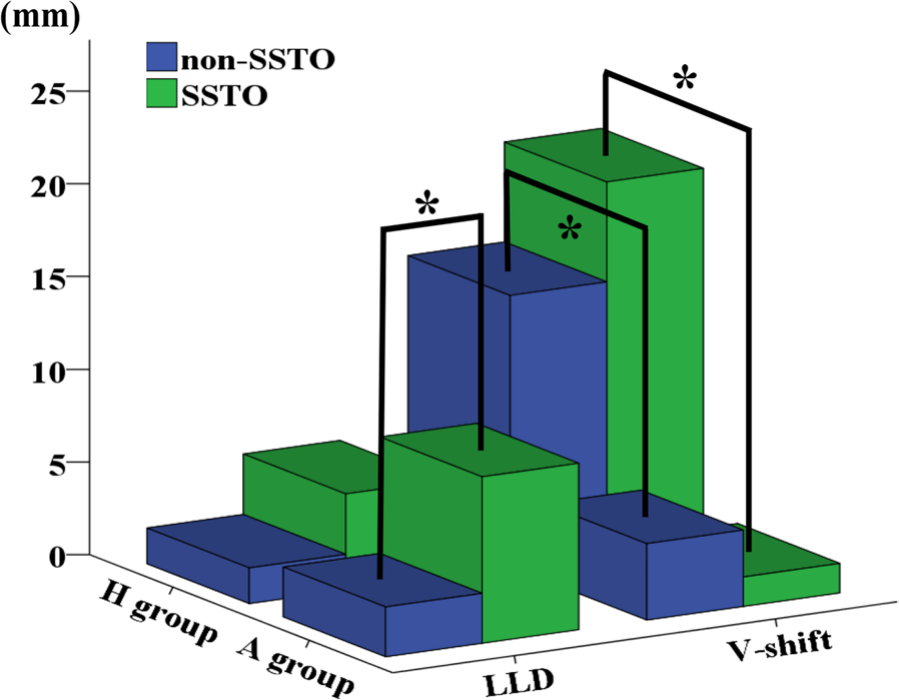
Comparison of LLD and V-shift based on the presence and absence of SSTO in both groups. * *p* < 0.05

**Fig. 4:**
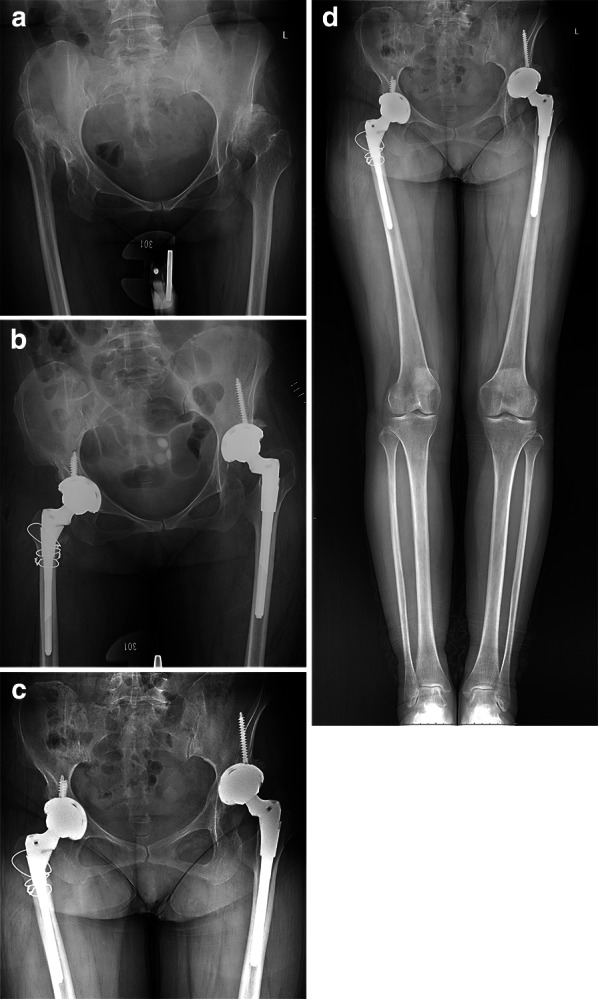
Preoperative anterior–posterior X-ray highlighting a bilateral DDH (right hip as Crowe IV and left hip as Crowe III) in a 49-year-old female patient (**a**). Postoperative X-ray showing an asymmetrical reconstruction of the Crowe III hip with high hip center and the Crowe IV hip at the anatomical position (**b**). SSTO was performed in the Crowe IV hip, and the osteotomy length was 3.5 cm. At final follow-up, radiographic evaluation after 3.5 years showing no loosening (**c**). Full-length standing anteroposterior radiograph showing that both lower limbs were of equal length (**d**)

**Fig. 5:**
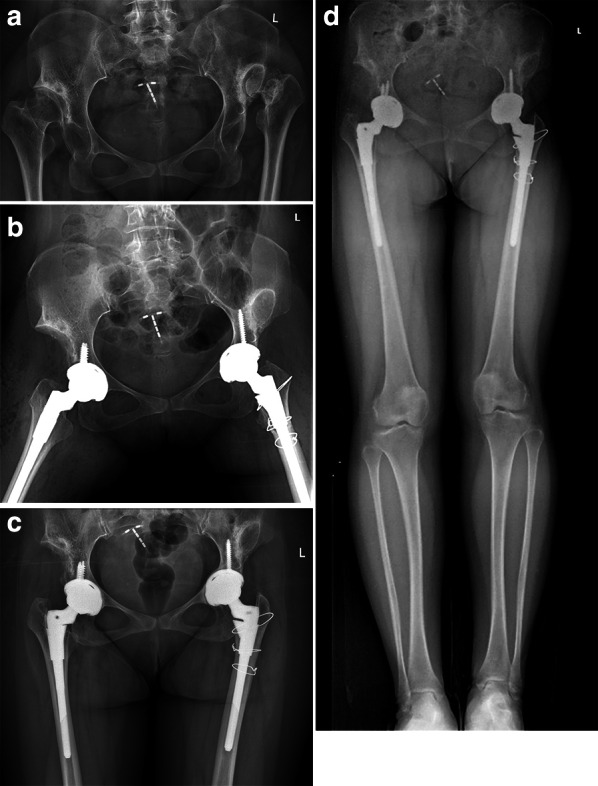
Preoperative anterior–posterior X-ray showing a bilateral DDH (right hip as Crowe III and left hip as Crowe IV) in a 51-year-old female patient (**a**). Postoperative X-ray showing bilateral anatomical reconstruction (**b**). SSTO was performed in the Crowe IV hip, and the osteotomy length was 2.0 cm. At final follow-up, radiographic evaluation after 1.8 years showing no loosening (**c**). Full-length standing anteroposterior radiograph showing that the right leg was 1.3 cm longer than the left leg (**d**)

Considering the cup position measurements, the mean V-COR and H-COR in the H group were 30.6 ± 5.8 mm (range 22.2–44.5 mm) and 30.0 ± 5.5 mm (range 21.7–41.3 mm), respectively. In the A group, the corresponding values were 14.0 ± 4.3 mm (range 7.0–21.1 mm) and 23.0 ± 2.3 mm (range 20.0–27.6 mm). A significant difference was found in terms of V-COR and H-COR between the two groups. The mean V-shift was 18.1 ± 7.6 mm (range 5.0–31.5 mm) in the H group and 2.8 ± 4.0 mm (range −2.5 to 10.6 mm) in the A group (*p* < 0.001). The mean H-shift was 7.9 ± 5.3 mm (range, −1.2 to 19.3 mm) in the H group and −0.3 ± 2.7 mm (range, −5.1 to 5.3 mm) in the A group (p < 0.001). In addition, there was no significant difference regarding the cup inclination, ALA, ALA ratio, and LLD between the two groups (Table 3). As illustrated in Fig. 2, there were three (13%) patients of the H group and four (21%) patients of the A group in LLD > 10 mm (*p* = 0.682). All seven patients in LLD > 10 mm underwent SSTO of the contralateral Crowe IV hip. Subgroup analysis based on the presence and absence of SSTO is presented in Table 4. No difference in mean osteotomy length between the two groups was found (*p* = 0.700). Both in the H group and A group, the LLD of SSTO group was greater than that of the non-SSTO group, but the difference was only statistically significant in the A group (Fig. 3). At final follow-up, no radiographic loosening was recorded in any patients (Figs. 4–5).

**Table 3:** Postoperative radiographic measurements

Parameter	H group	A group	*p* value
V-COR (mm)	30.6 ± 5.8 (22.2–44.5)	14.0 ± 4.3 (7.0–21.1)	< 0.001
V-shift (mm)	18.1 ± 7.6 (5.0–31.5)	2.8 ± 4.0 (−2.5 to 10.6)	< 0.001
H-COR (mm)	30.0 ± 5.5 (21.7–41.3)	23.0 ± 2.3 (20.0–27.6)	< 0.001
H-shift (mm)	7.9 ± 5.3 (−1.2 to 19.3)	−0.3 ± 2.7 (−5.1 to 5.3)	< 0.001
Inclination (degree)	38.4 ± 6.5 (29.0–49.5)	37.4 ± 6.8 (27.0–53.3)	0.648
ALA (mm)	50.5 ± 8.1 (32.9–66.0)	50.5 ± 4.8 (39.8–61.8)	0.993
ALA ratio	1.02 ± 0.20 (0.60–1.52)	1.11 ± 0.15 (0.85–1.44)	0.160
LLD (mm)	4.1 ± 6.6 (−5.8 to 19.7)	6.0 ± 7.0 (−3.2 to 21.1)	0.362

V-COR, vertical center of rotation; H-COR, horizontal center of rotation; ALA, abductor level arm; LLD, leg length discrepancy

**Table 4:** Comparison of LLD and V-shift based on the presence and absence of SSTO in both groups

	H group			A group			*p* value^a^	
	SSTO	Non-SSTO	*p* value	SSTO	Non-SSTO	*p* value	SSTO	Non-SSTO
LLD	5.2 ± 7.1	1.9 ± 5.3	0.271	9.0 ± 8.4	2.7 ± 2.8	0.048	0.238	0.712
V-shift	20.0 ± 7.7	14.6 ± 6.3	0.105	1.6 ± 4.2	4.1 ± 3.4	0.171	< 0.001	0.001

^a^Difference between the H group and A group in the SSTO and non-SSTO; SSTO, shortening subtrochanteric transverse osteotomy; LLD, leg length discrepancy

### Clinical evaluation

There was no revision during the follow-up period in either group. Compared with preoperative values, the mean HHS at the last follow-up showed a significant improvement in both groups. In the H group, the mean HHS improved from 52.5 ± 8.8 points to 90.0 ± 5.7 points (*p* < 0.001). In the A group, it improved from 54.5 ± 14.3 points to 92.4 ± 4.4 points (*p* < 0.001). No significant difference was found in the postoperative HHS between the two groups (*p* = 0.132). Only two patients in the H group and one patient in the A group presented with a positive Trendelenburg sign. In addition, in the H group, four patients showed a slight limp and three patients showed a moderate limp. Of these seven patients, four patients showed knee valgus deformity in the side of Crowe IV hip, which was absent before the surgery. In the A group, six patients presented with a slight limp and one patient with a moderate limp. Bilateral knee valgus deformity was observed in three patients. In the A group, one patient had femoral nerve palsies with numbness of the medial aspect of the lower limb. This patient recovered completely 1 year after the surgery. In the H group, patient satisfaction was reported as excellent for 10 patients, good for 11 patients, moderate for 2 patients, and unsatisfactory for 0 patients. In the A group, the corresponding numbers were 11, 7, 1, and 0 (*p* = 0.350).

## Discussion

High hip center technique has become a prevalent method to address the problem of segmental deficiency in Crowe II–III DDH hips. The midterm results of this technique have been encouraging in recent reports [7, 8, 10]. However, for patients with bilateral dysplasia who have asymmetrical reconstruction of two hips, the outcomes are still unknown. Therefore, in this study, we aimed to evaluate the functional and radiographical results of asymmetrically reconstructed THA in patients with bilateral dysplastic arthritic hips with one hip Crowe II–III and the other Crowe IV. For comparison, we included bilateral DDH patients with one Crowe IV hip and the other dysplastic hip both reconstructed in the anatomical position as the anatomic group.

Asymmetrical reconstruction was mainly manifested by the difference in the cup position of the bilateral hips. Our radiographic measurements showed that, in the H group, both the V-COR and the V-shift were significantly higher than those of the A group. A significant difference was also observed in terms of the H-COR and H-shift between the two groups. The H-shift of the H group indicated the relatively lateral placement of HHC hip compared with the contralateral Crowe IV hip. This might be partly due to the Crowe IV hip having been reconstructed in the inferomedial position instead of the real anatomic location, making the bilateral asymmetry more significant [13].

Many authors have reported previously the adverse effect of lateral placement on clinical outcomes and, consistently, these researchers all emphasized the importance of medialization in HHC reconstruction [18, 19]. In our study, the H-COR of the H group (30.0 ± 5.5 mm) was comparable to the results described by previous studies, such as 30.4 mm described by Flecher et al. [20] and 31.6 mm described by Galea et al. [7]. In a retrospective study of 85 HHC hips, Shen et al. [8] observed 12 acetabular cups with lateral placement greater than 10 mm compared with the anatomical center; however, no complications such as loosening and liner wear occurred with excessive lateralization. In this study, we also did not find any radiographic loosening or significant wear. Previously, Fukui et al. [12] found that if the FO and the ALA position were restored, hip abductor strength can be properly maintained. In our study, despite the superolateral placement of the acetabular cup in HHC reconstruction, there was no difference in the ALA between the two groups and the ALA ratio was approximately restored to 1:1. Thus, these radiographic results indicated that the high center of rotation in bilateral asymmetrical reconstruction of two hips was acceptable in this study.

The restoration of LLD is a major problem during asymmetrical reconstruction. Both the elevation of the HHC hip and the SSTO of the contralateral Crowe IV hip brought big challenges to the restoration of LLD. Recent research of gait analysis in leg length discrepancy-differentiated hip replacement DDH patients has revealed that LLD < 10 mm could provide a similar kinematic result between two limbs [21]. In this study, the mean LLD was 4.1 ± 6.6 mm in the H group and 6.0 ± 7.0 mm in the A group (*p* = 0.362), and there were only three (13%) patients of the H group and four (21%) patients of the A group with LLD > 10 mm (*p* = 0.682). Interestingly, all these patients who had LLD > 10 mm underwent SSTO of the contralateral Crowe IV hip. Subgroup analysis was then performed on the basis of the presence or absence of SSTO. We found that both in the H group and the A group, the LLD of SSTO group was greater than that of the non-SSTO group, and a significant difference was observed in the A group. This indicated that SSTO led to femoral shortening on the Crowe IV side, thus preventing the restoration of LLD. Similarly, of 36 SSTO patients with unilateral Crowe IV DDH, Du et al. [14] reported 10 patients with LLD > 10 mm and concluded that SSTO has negative effects on postoperative LLD. Wang et al. [22] also found postoperative LLD 10–20 mm in 39% of Crowe IV DDH patients with the use of SSTO. However, for a high dislocation hip with soft tissue contracture, to reduce the femoral head into the true acetabular position without excessive soft tissue tension and sciatic nerve injuries, SSTO was sometimes inevitable, even if that left the leg shortened and made the restoration of LLD more difficult [23]. Besides, it was notable that despite a greater LLD in SSTO patients of the H group, no significant difference was shown between the SSTO and non-SSTO group. One possible explanation was that the elevation of the HHC hip was to some extent a compensation to the SSTO of the contralateral side and reduced the LLD.

In this study, we found a high incidence of postoperative limp, 30.4% (7 patients) in the H group and 36.8% (7 patients) in the A group. Of these seven patients of the H group, four patients showed knee valgus deformity in the side of Crowe IV hip, and bilateral knee valgus deformity was observed in three patients of the A group. Thus, with the exception of leg length discrepancy, we think that the knee valgus deformity, which was a common complication after THA in patients with Crowe IV DDH and might be caused by excessively tight soft tissue, is another probable explanation for postoperative abnormal gait [24]. Besides, compared with the high hip center, anatomical reconstruction lengthened the lower limb more significantly, thus increasing the possibility of knee valgus.

There are several limitations to the present study. First, this study had a relatively small sample size. There were only 23 patients in the H group and 19 patients in the A group. However, it is uncommon for a patient with bilateral dysplasia to have reconstruction of one Crowe II-III hip with high hip center and the other Crowe IV hip at the anatomical position. Second, the A group included Crowe I, III, and IV hips, while there were only II and III hips in the H group. However, the percentage of Crowe I and IV in the A group was only 31.6%. Third, the follow-up time was short- to mid-term. Nevertheless, as the first study focusing on patients with bilateral dysplasia who have asymmetrical reconstruction of two hips, it is still of great value.

In conclusion, based on the clinical and radiographic evaluation in this study, asymmetrical reconstruction in patients with bilateral dysplastic arthritic hips with one hip Crowe II–III and the other Crowe IV is acceptable and comparable when compared with bilateral anatomical reconstruction. However, a study with larger sample size and longer follow-up time is required.

## Data Availability

The data will be made available by the authors upon reasonable request.

## Abbreviations

THA: Total hip arthroplasty
DDH: Developmental dysplasia of the hip
HHC: High hip center
LLD: Leg length discrepancy
SSTO: Shortening subtrochanteric osteotomy
V-COR: Vertical center of rotation
H-COR: Horizontal center of rotation
V-shift: Vertical shift
H-shift: Horizontal shift
ALA: Abductor lever arm
HHS: Harris hip score
